# Supplementary material for: Development and evaluation of a machine learning-based in-hospital COVID-19 disease outcome predictor (CODOP): A multicontinental retrospective study
Source: eLife. 2022 May 17;11:e75985. doi: 10.7554/eLife.75985 (PMC9129872; doi:10.7554/eLife.75985)
Supplement: Transparent reporting form [file elife-75985-transrepform1.docx]

***eLife’s* transparent reporting form**

We encourage authors to provide detailed information *within their submission* to facilitate the interpretation and replication of experiments. Authors can upload supporting documentation to indicate the use of appropriate reporting guidelines for health-related research (see [EQUATOR Network](http://www.equator-network.org/%20)), life science research (see the [BioSharing Information Resource](https://biosharing.org/)), or the [ARRIVE guidelines](http://www.plosbiology.org/article/info:doi/10.1371/journal.pbio.1000412) for reporting work involving animal research. Where applicable, authors should refer to any relevant reporting standards documents in this form.

If you have any questions, please consult our Journal Policies and/or contact us: [editorial@elifesciences.org](mailto:editorial@elifesciences.org).

**Sample-size estimation**

- You should state whether an appropriate sample size was computed when the study was being designed
- You should state the statistical method of sample size computation and any required assumptions
- If no explicit power analysis was used, you should describe how you decided what sample (replicate) size (number) to use

Please outline where this information can be found within the submission (e.g., sections or figure legends), or explain why this information doesn’t apply to your submission:

Sample-size estimation was not possible in our study. Our datasets are from all the COVID-19 patients that arrived to the more than 110 hospitals included in this study.

As indicated in the manuscript (please, see pages 2, 3, 5, and Figure 1 of the *Klen etal_Main Text and Main Figures* file) we analyzed clinical data from six different cohorts totaling 29223 COVID-19 patients treated in more than 150 hospitals in Spain, USA, Honduras, Bolivia, and Argentina, during three different pandemic waves extending from February 2020 to February 2022.

For the training, testing and validation of CODOP we divided the different patient cohorts either as time-sliced cohorts/datasets (in order test if the performance metrics of CODOP would strongly depend on the inherent changes occurring during the pandemic - e.g. the appearance of different virus variants, the used of more tailored clinical interventions), or by following geographical criteria (for the external test and validation with the USA and Latin American cohorts, respectively). Detailed description can be found in pages 5 and 7 of the *Klen etal_Main Text and Main Figures* file.

CODOP satisfies the Transparent Reporting of a multivariable prediction model for Individual Prognosis Or Diagnosis principles (TRIPOD; *Supplementary Table 15*), follows the recently proposed MINimum Information for Medical AI Reporting (MINIMAR; *Supplementary Table 16 and 17*), and it has been successfully checked for the risk of bias and applicability using the Prediction model study Risk of Bias Assessment Tool (PROBAST; *Supplementary Table* *18*).

**Replicates**

- You should report how often each experiment was performed
- You should include a definition of biological versus technical replication
- The data obtained should be provided and sufficient information should be provided to indicate the number of independent biological and/or technical replicates
- If you encountered any outliers, you should describe how these were handled
- Criteria for exclusion/inclusion of data should be clearly stated
- High-throughput sequence data should be uploaded before submission, with a private link for reviewers provided (these are available from both GEO and ArrayExpress)

Please outline where this information can be found within the submission (e.g., sections or figure legends), or explain why this information doesn’t apply to your submission:

Please, find the explanation for the division of the different patient cohorts used in this study in the previous section.

Missing values are characteristic for real-world clinical practice (perhaps more pronounced during a pandemic emergency) and may add distortion to any prediction model, therefore limiting the generalizability.

We have considered only variables with at most 40% of missing values. The percentage of missing values is listed in *Supplementary Table 1* of the *Klen etal_Supplementary Material* file. Most of the variables have less than 5% of missing values. We used mean for imputation because median values are very close to the values of the Survival group (due to the small proportion of deaths). The description of this imputation strategy can be found in page 5 of the *Klen etal_Main Text and Main Figures* file.

**Statistical reporting**

- Statistical analysis methods should be described and justified
- Raw data should be presented in figures whenever informative to do so (typically when N per group is less than 10)
- For each experiment, you should identify the statistical tests used, exact values of N, definitions of center, methods of multiple test correction, and dispersion and precision measures (e.g., mean, median, SD, SEM, confidence intervals; and, for the major substantive results, a measure of effect size (e.g., Pearson's r, Cohen's d)
- Report exact p-values wherever possible alongside the summary statistics and 95% confidence intervals. These should be reported for all key questions and not only when the p-value is less than 0.05.

Please outline where this information can be found within the submission (e.g., sections or figure legends), or explain why this information doesn’t apply to your submission:

The specifics for each of the statistical analysis used during development, testing and validation of CODOP can be found in the *Methods section* of the *Klen etal_Main Text and Main Figures* file, as well as, in the *Klen etal_Supplementary Material* file.

(For large datasets, or papers with a very large number of statistical tests, you may upload a single table file with tests, Ns, etc., with reference to sections in the manuscript.)

**Group allocation**

- Indicate how samples were allocated into experimental groups (in the case of clinical studies, please specify allocation to treatment method); if randomization was used, please also state if restricted randomization was applied
- Indicate if masking was used during group allocation, data collection and/or data analysis

Please outline where this information can be found within the submission (e.g., sections or figure legends), or explain why this information doesn’t apply to your submission:

Please, find the explanation for the division of the different patient cohorts used in this study in the first section of this document.

CODOP was built using modified stable iterative variable selection (SIVS) and linear regression with least absolute shrinkage and selection operator (lasso). The details can be found in page 5 of the *Klen etal_Main Text and Main Figures* file.

**Additional data files (“source data”)**

- We encourage you to upload relevant additional data files, such as numerical data that are represented as a graph in a figure, or as a summary table
- Where provided, these should be in the most useful format, and they can be uploaded as “Source data” files linked to a main figure or table
- Include model definition files including the full list of parameters used
- Include code used for data analysis (e.g., R, MatLab)
- Avoid stating that data files are “available upon request”

Please indicate the figures or tables for which source data files have been provided:

The raw patient data used in this study are not freely available due to legal restrictions imposed by the ethical committees of the different hospitals. However, they can be accessed upon request to the Scientific Committees of these organisms. An exception to this is the patient data from the USA cohort, which has been published elsewhere. We described this on page 11 of the Klen etal_Main Text and Main Figures file.

However, we now provide a .csv file with all the model’s numerical output necessary used by us to generate Figures 2, 3, and 4.

Furthermore, all supplementary tables can be found in the Klen etal_Supplementary Material file.

The final model can be found in the Klen etal_Supplementary Material file and it is freely accessible in the following Github addresses:

<https://github.com/TUC-Circular-Economy-Department/COvid-19-Disease-Outcome-Predictor#uir>

<https://github.com/TUC-Circular-Economy-Department/COvid-19-Disease-Outcome-Predictor#documentation>

The different R or Python code packages used in this study are free of use and indicated in the Methods section of the Klen etal_Main Text and Main Figures file.
